# Synthesis of PNA Oligoether Conjugates

**DOI:** 10.3390/molecules19033135

**Published:** 2014-03-13

**Authors:** Alice Ghidini, Peter Steunenberg, Merita Murtola, Roger Strömberg

**Affiliations:** 1 Department of Biosciences and Nutrition, Karolinska Institutet, Novum, Hälsovägen Huddinge 7, 14183, Sweden; E-Mails: alice.ghidini@ki.se (A.G.); merita.murtola@ki.se (M.M.); 2 ICL-IP Terneuzen, Frankrijkweg BJ, Terneuzen 6 4538, The Netherlands; E-Mail: SteunenbergP@icl-ip.com; 3 Department of Chemistry, University of Turku, Turku 20014, Finland

**Keywords:** peptide nucleic acid, polyethyleneglycol, aminosugar, neocuproine

## Abstract

Several different approaches have been explored for conjugation of oligoethers to PNA with internally or N-terminal placed diaminopropionic acid residues. Single and double conjugation of 2-(2-(2-aminoethoxy)ethoxy)ethanol was obtained using carbonyldimidazole. Using a post PNA-assembly coupling procedure the building block 2-(2-(2-(benzoyloxy)ethoxy)ethoxy)acetic acid multiple attachment of 2-(2-(2-hydroxyethoxy)ethoxy)acetyl groups to both N-terminal and β-amino groups of inserted diaminopropionic acids residues was achieved. Use of a new oligoether functionalized amino acid allows inclusion of oligoether conjugates during on-line machine assisted synthesis which also allowed combination of methods for attachment of different oligoethers and co-conjugation of neocuproine as well as conjugation of an aminosugar.

## 1. Introduction

Peptide nucleic acid (PNA) as first invented [1] are essentially N-(aminoethyl)glycine peptides carrying nucleic acid bases (aeg-PNA). PNA hybridizes to both DNA and RNA with Watson-Crick base pairing, thus mimicking the action of natural nucleic acids [2]. A variety of modifications of the original backbone of PNA have been reported [3,4] and numerous studies directed at diagnostic use as well as consideration for therapeutics, has occurred over the past two decades [5,6]. It is not uncommon for peptides, proteins, nanoparticles, oligonucleotides and even low molecular drugs to be conjugated to polyethyleneglykol (PEG) moieties in order to enhance uptake, solubility, stability, pharmacokinetics *etc.* for enhanced drug delivery [7,8]. Conjugates to PNA typically involves conjugation to the carboxy or amino terminals either as exemplified for nanoparticle applications [9,10] or a nucleic acids delivery construct [11]. Conjugation of shorter oligoethers to PNA is in general also to the carboxy or N terminal when the oligoether serves as a linker to other functionalities as in microRNA targeting PNA conjugated to a cellpenetrating peptide [12] or to the cleaver entity in a microRNA targeting PNA-based artificial nuclease [13]. An interesting example of backbone inclusion of an oligoether into a peptide nucleic acid is γ-PNA, which is a modification that involves [2-(2-methoxyethoxy)ethoxy]methyl branching of the aminoethyl moiety of aeg-PNA. This backbone modification enhances affinity for the target nucleic acid as well the solubility of the PNA in aqueous media [14].

**Figure 1:**
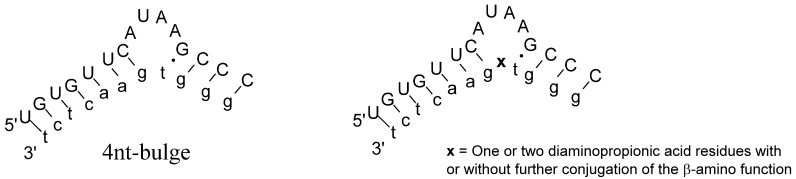
**Left** panel: A tetranucleotide RNA-bulge (upper case letters) formed by incomplete base pairing upon binding to a partially complementary PNA-strand (lower case letters). **Right** panel: A tetranucleotide RNA-bulge (upper case letters) formed upon binding to a partially complementary PNA-strand with one or two amino acids (**x**) incorporated.

When a duplex between two oligonucleotides contains one or several unpaired nucleotides in an internal part of one of the strands a bulge is formed (Figure 1). The occurrence of one or several bulged out nucleotides in an otherwise complementary duplex usually results in substantial destabilization of the complex [15]. Complexes with bulged out nucleotides can be stabilized by inclusion of modified nucleosides [16] or conjugation to entities that interact with the unpaired region [17]. Bulged RNA is also known to be more susceptible to cleavage than RNA in a helical structure and has been interesting as targets for 2'-O-methyloligonucleotide artificial nucleases (OBAN’s) [18,19,20] as well as PNA-based artificial nucleases (PNAzymes) [21,22].

In the current study we present an investigation on the conjugation of oligoethers to PNA with internally or N-terminal placed diaminopropionic acid residues. We were concerned how the addition of oligoethers may affect the binding of a target RNA and other properties of the PNA. In particular, we were interested to see if different oligoether constructs would hamper formation of the complex between the PNA conjugate and RNA that forms a non-paired bulge region (Figure 1) and which constructs that can be accepted in future artificial nuclease designs. Therefore several different methods and constructs were developed as reported below. In addition co-conjugation of neocuproine that has been used in artificial nucleases [18,19,20,21,22] is performed as well as conjugation of an aminosugar. 

## 2. Results and Discussion

The different approaches for the conjugation of oligoethers to PNA containing diaminopropionic acid (Dapa) were all performed with the PNA remaining on the solid support, where it was first assembled using Fmoc-chemistry. Prior to the post-conjugation, a 4-methyltrityl (Mtt) protecting group on the β-nitrogen of Dapa and/or the terminal Fmoc was removed. The methyltrityl (Mtt) group is acid-labile and readily removed by mild acidic treatment [23] and the amine is *in situ* neutralized in the subsequent conjugation reaction by adding an excess of base, such as N-Methylmorpholine (NMM) or *N*,*N*-Diisopropylethylamine. 

**Scheme 1:**
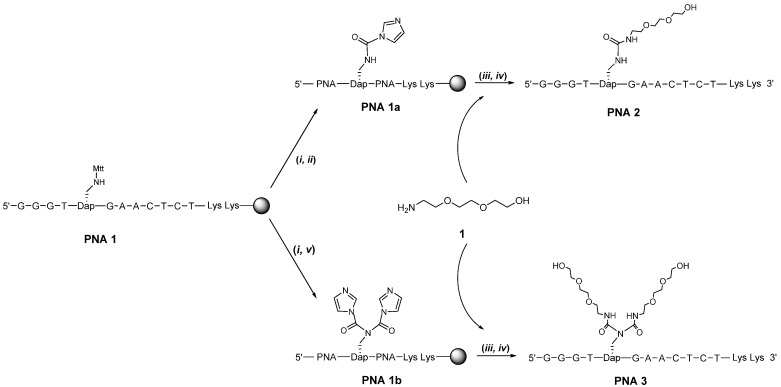
Synthesis of **PNA 2** and **PNA 3**.

First evaluated was a quick method that only involves commercial reagents, *i.e.*, conjugation by reacting 1,1-carbonyldiimidazole (CDI, in a similar fashion as developed for oligonucleotide peptide conjugates [24]) with the Dapa β-amino group to produce intermediate **PNA 1a** and then add the amino-oligoether **1** to substitute the imidazole ring of the intermediate (Scheme 1). The reaction was not that effective but the product **PNA 2** could be isolated by HPLC purification. Due to the incomplete conversion of **PNA 1** we performed double treatment with CDI, reducing the amount of unreacted PNA but the amount of **PNA 2** was not increased, instead a second product was formed that corresponds to the double substituted product **PNA 3** (Scheme 1) which also could be isolated through HPLC purification.

(*i*) 1% Trifluoroacetic acid (TFA) in DCM, 5 times for 1 min; (*ii*) CDI in N-Methyl-2-pyrrolidone (NMP), NMM, 1 h; (*iii*) oligoether **1**; (iv) TFA/Triisopropylsilane (TIS)/H_2_O (95/2.5/2.5) for 2 h; (*v*) CDI in NMP, NMM, 1 h, 2 times.

The CDI coupling procedure did produce both a mono and double conjugation product. However, the limited conversion, and somewhat demanding purification, made this procedure less recommendable. This suggested to us that we should look at other methodologies as well. Nevertheless **PNA 2** and **PNA 3** were isolated so that we could evaluate if the stability of the complex with RNA that forms a bulge upon hybridization to the PNA (Figure 1, right panel) is much affected. Thermal melting analysis with the target sequence UGUGUUCAUAAGCCC revealed that the T_m_ values for the complexes was 49 °C for both **PNA 2** and **PNA 3**. Although this is somewhat lower compared to the complex with the non-conjugated PNA (T_m_ = 54 °C) of the same sequence, the oligoethers seem to be accommodated at a reasonable trade-off of T_m_ and not interfering so severely that it would prevent association of the PNA with the RNA. 

**Scheme 2:**
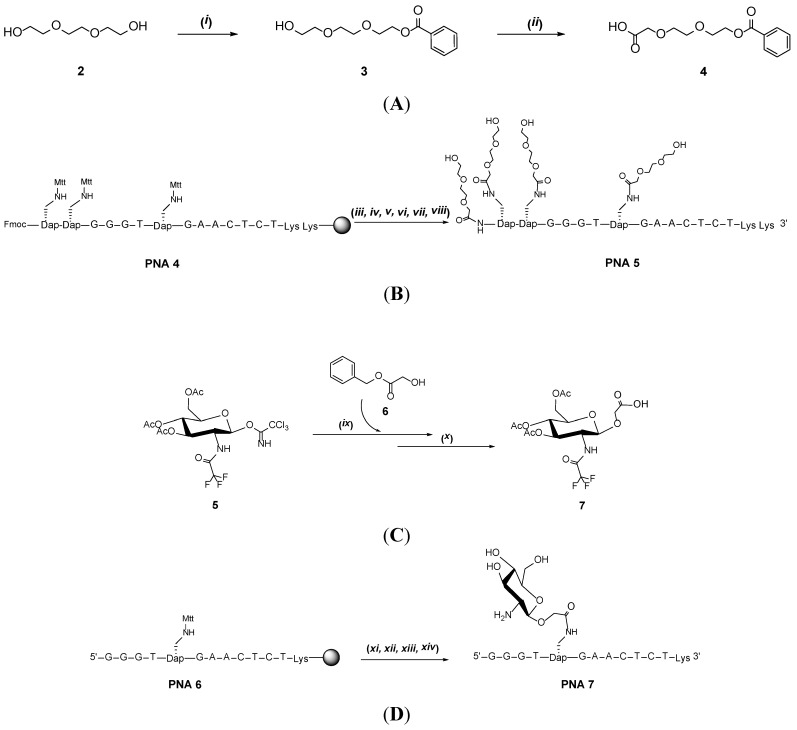
Synthesis of oligoether building block **4** (**A**), tetraoligoether PNA conjugate **PNA 5** (**B**), aminosugar **7** (**C**) and PNA-aminosugar conjugate **PNA 7** (**D**).

To get a method for postconjugation that was not only cleaner but also would allow multiple conjugations we then decided to utilize conditions similar to those for peptide synthesis with *N*,*N*,*N*′,*N*′-tetramethyl-O-(1H-benzotriazol-1-yl)uronium hexafluorophosphate (HBTU)/1-hydroxybenzotriazole hydrate (HOBt) and an oligoether carrying a carboxylate function that can be coupled to the β-amino group of Dapa and/or the N-terminal amine. To obtain conjugates with a terminal hydroxyl group on the oligoether, compound **4** was synthesized by protecting one terminal of triethylene glycol with a benzoyl group and oxidizing the other hydroxyl group to a carboxylic acid (Scheme 2A). **PNA 4** was synthesized and the Mtt groups removed with the PNA still on support whereupon coupling of oligoether **4** was performed by a procedure similar to that used for coupling of amino acids to the Dapa side chain [17,25], but with reduced excess of reagent (Scheme 2B). One could remove both Fmoc and Mtt protection before conjugation to obtain the fully oligoether conjugated PNA 5. However, to verify that orthogonal functionalization could be pursued we performed the side-chain and α-amino conjugation in separate steps.

In order to debenzoylate the oligoether the solid support was treated with 20% NH_3_/MeOH solution at room temperature overnight. However, after release of the PNA from the support the chromatogram and mass spectrum of the crude material revealed that debenzoylation was incomplete. Additional deprotection of the crude material with methanolic ammonia at 50 °C for 5 h gave complete conversion and **PNA 5** was isolated by HPLC. Also for this PNA oligoether conjugate there was an even smaller effect of the conjugation of the four oligoether groups on the stability of the complex formed with the RNA sequence UGUGUUCAUAAGCCC, than found for the products obtained with the CDI reaction (see above). Thermal melting studies gave a T_m_ of 51 °C for the RNA complex with **PNA 5** (T_m_ for the RNA complex with non-conjugated PNA = 54 °C) which suggests that there is less negative interference with the PNA/RNA complex than with the CDI linked hydroxyoligoether.

(**A**) (*i*) Triethylene glycol: Benzoyl chloride 3:1, pyridine; (*ii*) Acetone, H_2_SO_4_ (1.5 M), Cr_2_O_3_; (**B**) (*iii*) 20% Piperidine in NMP; (*iv*) oligoether 4, HBTU, HOBt, NMM; (*v*) 1% TFA in DCM, 5 times for 1 min; (*vi*) oligoether 4, HBTU, HOBt, NMM; (*vii*) 20% NH_3_/MeOH, 5 h, 50 °C; (*viii*) TFA/TIS/H2O (95/2.5/2.5), 2 h; (**C**) (*ix*) protected glycolic acid 6, dry CH_2_Cl_2_, TMSOTf, from −20 °C to room temperature, 3 h; (*x*) Pd-C catalyst in THF-MeOH (1:1), overnight; and (**D**) (*xi*) 1% TFA in DCM, 5 times for 1 min; (*xii*) Aminosugar **5**, HATU, DIPEA in NMP, 1h; (*xiii*) TFA/TIS/H2O (95/2.5/2.5) for 2 h; (*xiv*) Ammonia, 5 h, 50 °C.

Another class of compounds that can be interesting to conjugate to PNA, not only for increase of aqueous solubility, is sugars and aminosugars. As an example of this, and to evaluate if PNA/RNA complex with a bulge would be affected much by the presence of sugar, we performed a conjugation to a 2-aminoglucose derivative. Compound **5** was synthesized as described with initial protection of the amino group and successive acetylation of hydroxyl groups and adding a trichloroacetimidate group in the anomeric position [26,27,28]. Subsequent glycosidation with a protected glycolic acid then gave product **7** [29,30]. Reacting solid supported **PNA 6 **with an excess of compound **7** (30 eq) and HATU as condensing agent allowed us to obtain the aminosugar conjugate **PNA 7**. Thermal melting studies gave a T_m_ of 51 °C for the RNA complex with **PNA 7** (T_m_ for the RNA complex with non-conjugated PNA = 54 °C).

**Scheme 3:**
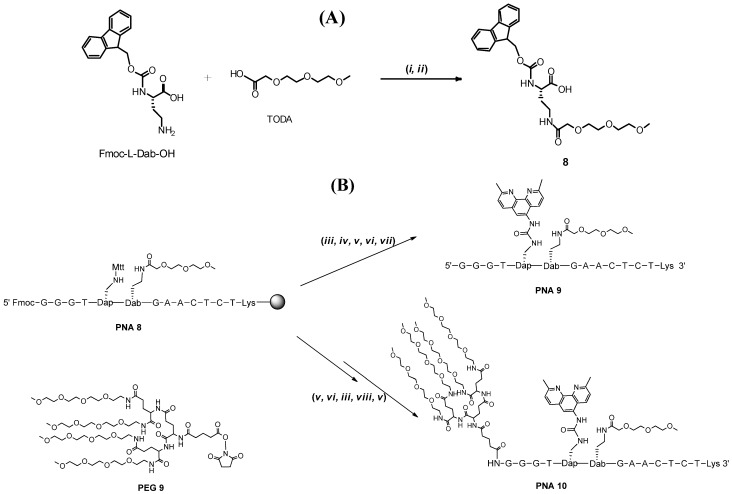
Synthesis of amino acid **8** (**A**), neocuproine and oligoether conjugate **PNA 9** and **PNA 10** (**B**).

In order to allow for more ready orthogonal conjugation of different groups we then turned to an alternative to post conjugation, where internal incorporation of an oligoether building block into PNA was evaluated. If this on-line approach is combined with the post-conjugations it would allow incorporation of three different conjugated entities, even though only Mtt and Fmoc temporary protection is used. To accomplish on-line incorporation we synthesized an oligoether carrying amino acid building block that can be inserted in the PNA sequence on the automated synthesizer. At the same time we also wished to see if a methoxy terminated oligoether would be accepted in PNA/RNA bulge complex. Thus, the commercially available oligoether 2-[2-(2-methoxyethoxy)ethoxy]-acetic acid (TODA) (Scheme 3A), which carries a terminal O-methyl group, was coupled with of HATU-preactivated Fmoc-L-Dab-OH (N-α-Fmoc-L-2,4-diaminobutyric acid) to obtain the oligoether amino acid building block **8**. The amino acid **8** was then used in the synthesis of **PNA 9** that is doubly conjugated with both an oligoether and neocuproine, which in the presence of a metal ion such as Zn^2+^ [18,19,20,21] or Cu^2+ ^[22] turns into an RNA cleaver. The synthesis could be achieved by a standard PNA synthesis protocol on an automated synthesizer followed by removal of the Mtt from the Dapa unit and then post conjugation with 5-phenoxycarbonylaminoneocuproine [18,19] followed by removal of Fmoc, capping, cleavage from support and deprotection. Thermal melting studies gave a T_m_ of 52 °C for the RNA complex with **PNA 9** (T_m_ for the RNA complex with non-conjugated PNA = 54 °C). Thus, an oligoether in this position can be acceptable in future artificial nuclease designs without substantial compromise with regard to the stability of the PNA/RNA bulge complex.

(**A**) (i) Preactivation of TODA with HATU, NMM in DMF (2 min); (ii) Fmoc-L-Dab-OH; (**B**) Synthesis of **PNA 9 **and **PNA 10**: (iii) 20% Piperidine in NMP, 30 min; (iv) acetic anhydride: lutidine: NMP 5:6:89 2 times for 5 min; (v) 1% TFA in DCM, 5 times for 1 min; (vi) 5-PhOC(O)NH-Neocuproine, NMM in NMP overnight reaction; (vii) TFA/TIS/H2O (95/2.5/2.5) for 2 h; (viii) PEG 9, NMM, NMP.

We then wished to investigate if extensive multiple oligoether conjugation at the N-terminal would influence the stability of the complex and since the N-terminal Fmoc is orthogonal, additional complexity can be achieved by also conjugating at the amino terminal position. Thus, **PNA 8** was converted to **PNA 10** by reaction of the PNA with the reagent **PEG 9** after removal of the Fmoc. **PNA 10** was obtained but with this triply conjugated derivative carrying one neocuproine and five oligoether chains it was apparent that the solubility in water was problematic (to the extent that higher percentage of acetonitrile was necessary for the HPLC purification) and hence reliable thermal melting with the RNA complement could not be obtained. It appears that just addition of more oligoether chains does not necessarily give improved aqueous solubility. 

In order to see if we could make sense of how the polarity was affected by oligoether conjugation we compared the RP-HPLC retention times for the conjugates and a couple of additional constructs (Table 1). **PNA-Dapa-PNA 1** and **PNA-Dapa-PNA 6** were obtained by complete deprotection of **PNA 1** and **PNA 6**. These are as **PNA 2** and **PNA 3** or as **PNA 7** respectively, but with a non-conjugated Dapa in the central position. In addition, **PNA-Gly-PNA** which is **PNA-Dapa-PNA 6** with a glycine instead of the Dapa and **PNA-GlyNeo-PNA** which is as **PNA 9** but with a glycine instead of the Dab-oligoether) without oligoethers were also synthesized. 

**Table 1:** RP-18 retention times for PNA conjugates.

Entry	PNA Construct	RP-18 HPLC Ret. Time (min) ^a^	Type and Number of Conjugated Entities
1	**PNA-Dapa-PNA 1**	13.7	1 Dapa
2	**PNA 2**	15.7	1 hydroxyoligoether
3	**PNA 3**	21.7	2 hydroxyoligoethers
4	**PNA 5**	21.1	1 central and 3 terminal hydroxyoligoethers
5	**PNA-Dapa-PNA 6**	17.7	1 Dapa
6	**PNA-Gly-PNA**	17.6	1 glycine
7	**PNA 7**	15.8	1 aminosugar
8	**PNA-GlyNeo-PNA**	22.1	1 glycine and 1 neocuproine
9	**PNA 9**	25.5	1 methoxyoligoether and 1 neocuproine
10	**PNA 10**	27.9 ^b^	1 methoxyoligoether and 1 neocuproine and 4 terminal methoxyoligoethers

^a^ Linear gradient of 0%–20% acetonitrile for 30 min. (with 0.1% TFA) on an Ascentis Express Supelco Peptide ES-C18 (2,7 µm 150 × 4.6 mm) column at 60 °C with a flow rate of 1 mL/min; ^b^ A higher percentage of acetonitrile was needed for this conjugate: Linear gradient of 7.5%–35% acetonitrile for 36 min. (with 0.1% TFA) on an Ascentis Express Supelco Peptide ES-C18 (2,7 µm 150 × 4.6 mm) column at 60 °C with a flow rate of 1 mL/min.

It is not an absolutely clear picture for these comparisons but conjugation of oligoethers seems to give a lower polarity, as judged by the shorter retention times during RP-HPLC analysis (*cf.* entries 1, 5, 6 and 8 with entries 2, 3, 4, 9 and 10). The only clear decrease of retention time, and presumably polarity, comes from conjugation of the aminoglucoside moiety (*cf.* entry 5–7).

## 3. Experimental

### 3.1. Materials and Methods

Peptide nucleic acid monomers were from Link Technologies Ltd. (Bellshill, UK). Rink Amide resin was purchased from Biotage (Uppsala, Sweden). HBTU, HOBt and diaminopropionic acid and derivatives were purchased from Novabiochem (now incorporated into Merck Millipore, Darmstadt, Germany). Solvents and reagents for solid- phase synthesis were synthesis grade from Applied Biosystems (now incorporated into Life Technologies Europe, Stockholm, Sweden) and IRIS Biotech Gmbh (Marktredwitz, Germany). **PEG 9** was purchased from Iris Biotech Gmbh. Other solvents were purchased from Merck Eurolab (Darmstadt, Germany). High-resolution mass spectrometry (HRMS) was performed on a Micromass LCT electrospray time-of-flight (ES-TOF) mass spectrometer in acetonitrile–water 1:1 (*v/v*) solutions. The molecular weights of the oligoribonucleotide and peptide nucleic acid conjugates were reconstructed from the *m/z* values using the mass deconvolution program of the instrument (Mass Lynx software package). The RNA substrate was purchased from Thermoscientific and was first purified by semi-preparative IE-HPLC (ion exchange high performance liquid chromatography) and then purified with RP-HPLC. Thermal melting analysis was determined from an absorbance *vs.* temperature profile measured at 260 nm on a Varian Cary 300 UV–vis dual beam spectrophotometer (Varian). Concentrations of both RNA and PNA were determined by UV absorption at 260 nm and calculated from extinction coefficients obtained by the nearest neighbor approximation [31]. All chemicals used in the kinetics experiments were of molecular biology grade.

### 3.2. Synthesis of PNAs

**PNA 1**, **PNA 4**, **PNA 6**, **PNA 8**, **PNA-Gly-PNA** and **PNA-GlyDapa-PNA** sequences were assembled automatically on a solid support (Rink Amide resin) using the manufacturer’s protocol for the Applied Biosystems 433A peptide synthesizer with 9-fluorenylmethyloxycarbonyl (Fmoc)-chemistry and HATU (1-[Bis(dimethylamino)methylene]-1H-1,2,3-triazolo[4,5-b]pyridinium 3-oxid hexafluorophosphate, from Iris Biotech) as coupling agent. PNA building blocks were from Link Technologies Ltd (Strathclyde, UK) and Fmoc-^α^N-Lys (^ε^N-Boc)OH was from Iris Biotech Gmbh (Marktredwitz, Germany). PNAs were purified with an Ascentis Express Supelco Peptide ES-C18 (2, 7 µm 150 × 4.6mm) column at 60 °C using a flow rate of 1 mL/min and a linear gradient of 40% B for 30 min. (A) 0.1% TFA–aq., (B) 0.1% TFA–aq., 50% MeCN.

**PNA-Dapa-PNA 1** and **PNA-Dapa-PNA 6** conjugates were obtained by cleaving **PNA 1** and **PNA 6, **respectively, from support by TFA/TIS/water (95/2.5/2.5) (200 µL) for 2 h. Products were freeze dried and purified on an Ascentis Express Supelco Peptide ES-C18 (2, 7 µm 150 × 4.6 mm) column at 60 °C using a flow rate of 1 mL/min and a linear gradient of 40% B for 30 min. (A) 0.1% TFA–aq., (B) 0.1% TFA–aq., 50% MeCN. (Retention time **PNA-Dapa-PNA 1**: 13.7 min and **PNA-Dapa-PNA 6**: 17.7 min). **PNA-Dapa-PNA 1**: C136H181N71O38 [M+], 3418; found, 3418 and **PNA-Dapa-PNA 6**: C129H166N68O37, 3259; found [M+] 3260.

**PNA-Gly-PNA **was obtained cleaving from support by TFA/TIS/water (95/2.5/2.5) (200 µL) for 2 h, freeze dried and purified with a Ascentis Express Supelco Peptide ES-C18 (2, 7 µm 150 × 4.6 mm) column at 60 °C using a flow rate of 1 mL/min and a linear gradient of 40% B for 30 min. (A) 0.1% TFA–aq., (B) 0.1% TFA–aq., 50% MeCN. (Retention time **PNA-Gly-PNA 1**: 17.6 min). **PNA-Gly-PNA**: C129H166N68O37, 3259; found [M+] 3260.

### 3.3. Post-Conjugation of Oligoethers to PNA

*Synthesis of **PNA 2** and **PNA 3**.* N^β^-methyltrityl protection was cleaved off by subjecting the solid support bound **PNA 1** (1. 4 µmol) to 1% trifluoroacetic acid (TFA) in dichloromethane (DCM) for 5 × 1 min, followed by washing with DCM and NMP. CDI (1, 1′-Carbonyldiimidazole) (0.014 mmol, 10 eq) was dissolved in NMP (150 µL) in a septum capped vial and NMM (4-Methylmorpholine) (0.071 mmol, 50 eq) was added to the solution. The mixture was added to the support bound **PNA 1** through a septum and the reaction mixture was agitated for 1 h. The support was filtered but not washed. Polyether 1 (0.71 mmol) was then dissolved in NMP (150 µL) and added to the same support with 40 µL of NMM and shaken overnight. The support was filtered and washed with NMP and DCM.

**PNA 3** was obtained when the CDI treatment was repeated twice and otherwise following the procedure for the oligoether **1** coupling as when isolating **PNA 2**.

The PNA conjugates were cleaved from support by TFA/TIS/water (95/2.5/2.5) (200 µL) for 2 h, freeze dried and purified with a Ascentis Express Supelco Peptide ES-C18 (2, 7 µm 150 × 4.6mm) column at 60 °C using a flow rate of 1 mL/min and a linear gradient of 40% B for 30 min. (A) 0.1% TFA–aq., (B) 0.1% TFA–aq., 50% MeCN. (Retention time **PNA 2**: 15.7 min and **PNA 3**: 21.7 min). **PNA 2**: C143H194N72O42 [M+], 3591; found, 3590 and **PNA 3**: C150H207N73O46, 3767; found [M+] 3769.

*Synthesis of 2-(2-(2-hydroxyethoxy)ethoxy)ethyl benzoate* (**3**). Triethylene glycol (3 mL, 3 eq, 30 mmol) was twice dried by evaporation of added pyridine (20 mL). The residue was dissolved in pyridine (40 mL) and benzoyl chloride (1.2 mL, 1 eq, 10 mmol) was then added dropwise to the dry solution at 0 °C and at an interval of 10 min. After complete addition, the solution was allowed to obtain room temperature and the reaction was monitored by TLC. After 2 h most of the reagent was converted and the reaction was stopped by adding 5 mL of water and the pyridine was evaporated in the presence of added acetonitrile. The crude material was dissolved in toluene and washed with NaHCO_3_ to remove excess of the diol and the organic phase was dried over magnesium sulfate. The product was isolated by flash chromatography on a column of silica gel using gradient hexane in ethyl acetate giving 1,02 g of **3** (40%). Mass calculated for compound 3, 254.12; found, [M+23(Na)] 277.46; 1H-NMR (CDCl3): δ 8.06 (d, 2H, *J* = 8); δ 7.56 (t, 1H, *J* = 7.4); δ 7.44 (t, 2H, *J* = 7.6); δ 4.50 (t, 2H, *J* = 4.7); δ 3.84 (t, 2H, *J* = 5.1); δ 3.87 (m, 6H); δ 3.61 (t, 2H, *J* = 4.6).

*Synthesis of 2-(2-(2-(benzoyloxy)ethoxy)ethoxy)acetic acid* (**4**). Compound **3** (0.30 g, 1.2 mmol) was dissolved in acetone (6 mL) and added dropwise to a solution of sulfuric acid (1.5 M, 7.2 mL) containing chromium (VI) oxide (0.41 g, 4.1 mmol) at 0 °C. After complete addition of the alcohol, the solution was kept at room temperature for 24 h. The chromium salts were removed by steps of filtrations and centrifugations and the clear solution was concentrated under reduce pressure. The crude material was extracted from the solution using toluene (3 × 40 mL) and dried over magnesium sulfate. After filtration and concentration, the crude oil product was purified using column chromatography on silica eluted with a gradient system running from pure dichloromethane with 1% acetic acid to dichloromethane 90% methanol 10% with 1% acetic acid. This gave the product as yellow oil (167 mg). Mass calculated for compound 4, 268.09; found, [M+23(Na)] 291.49; ^1^H-NMR (CDCl_3_): δ 8.06 (d, 2H, *J* = 7.5); δ 7.56 (t, 1H, *J* = 7. 5); δ 7.44 (t, 2H, *J* = 7.5); δ 4.52 (t, 2H, *J* = 3.9); δ 4.17 (s, 2H); δ 3.87 (t, 2H, *J* = 4.8); δ 3.76 (q, 2H, *J* = 5.4); ^13^C-NMR (CDCl3): δ 171.61.0; δ 165.55; δ 132.10; δ 128.89; δ 128.76; δ 127.39; δ 76.32; δ 76.00; δ 75.69; δ 67.61; δ 62.69.

*Synthesis of* **PNA 5**. The terminal Fmoc was cleaved off by subjecting **PNA 4** (4 mg, 1.13 µmol) to 20% piperidine in NMP for 25 min, followed by washing several times with DCM and NMP. Compound **4** (11.4 mg, 37.5 eq), HBTU (15.3 mg, 35.6 eq) and HOBt (5.4 mg, 35.6 eq) were dissolved in 97.5 µL of NMP and NMM (8.25 µL, 65 eq) and agitated for 5 min to preactivate the mixture, then added to the solid support and shaken for 30 min. The support was filtered and washed with NMP and DCM. The N^β^-methyltrityl protection of the diaminopropionic acid was removed by subjecting the solid support to 1% trifluoroacetic acid (TFA) in dichloromethane (DCM) for 5 × 1 min, followed by washing with DCM and NMP. Compound **4** (34.2 mg, 112.5 eq), HBTU (45.9 mg, 106.8 eq) and HOBt (16.2 mg, 106.8 eq) were dissolved in 318 µL of NMP and NMM (25.5 µL, 195 eq) were added before agitation for 5 min. The preactivated mixture was added to the support and shaken for 30 min. The support was filtered and washed with NMP and DCM. The PNA conjugate was deprotected using a 20% NH3/MeOH solution for 5 h at 50 °C and cleaved from support by TFA/TIS/water (95/2.5/2.5) (200 µL) for 2 h, freeze dried and purified as for **PNA 2** (Retention time **PNA 5**: 21.1 min). Mass calculated for **PNA 5**: C164H231N75O55, 4131; found, [M+] 4131.

*Synthesis of* (**7**). To a solution of anhydrous CH_2_Cl_2_ (3 mL), 4 Å mol. sieves (0.1 g), protected glycolic acid **6** (150 g, 0.91 mmol) and compound **5** (500 g, 0.91 mmol) was added, at −20 °C under argon atmosphere, TMSOTf (15 µL, 0,075 mmol). Afterwards the reaction mixture was stirred at this temperature for 3 h until TLC showed full conversion (PE:EtOAc 7:3, Rf 0.3). Than the mixture was quenched by the addition of Et_3_N, filtered and the residue washed with CH_2_Cl_2_ (100 mL). The organic layer was concentrated. Pd-C catalyst was added to a solution of the oily mixture obtained from the previous reaction in 75 mL of (1:1) THF-MeOH. Afterwards, under stirring, the reaction mixture was placed under an H_2_ gas atmosphere (atmospheric pressure) and the deprotection of the benzyl ester was continued overnight. When full conversion had taken place (TLC examination), the H_2_ flow was replaced by Argon and the reaction mixture was filtered and washed (MeOH, 200 mL) through a pad of Celite. Upon concentration of the filtrate, a white solid material was obtained, and added CH_2_Cl_2_ was evaporated to remove traces of MeOH to afford **7** as a white solid (274 g, 0.6 mmol, 65% yield). Mass calculated for compound **7**, 459.10; found [M−] 458; 1H-NMR (DMSO-d_6_): δ 6.26 (1H, NH); δ 5.18 (1H, t, *J* = 10); δ 4.81 (1H, t, *J* = 10); δ 4.73 (1H, d, *J* = 8.4) ; δ 4.15–4.04 (2H, m); δ 3.93 (2H, s, *J* = 2.4); δ 3.82–3.72 (2H, m); δ 1.98 (3H, s); δ 1.87 (3H, s); δ 1.83 (3H, s);. ^13^C-NMR (DMSO-d6): δ 171.1–169.3 (3 COCH3 and 1 COOH); δ 157.60; δ 114.2; δ 99.6; δ 77.0; δ 72.1; δ 70.6; δ 65.3; δ 62.0; δ 54.0; δ 20.68; δ 20.65; δ 20.48.

*Synthesis of* **PNA 7**. The N^β^-methyltrityl protection of the diaminopropionic acid was removed subjecting the solid support **PNA 6** to 1% trifluoroacetic acid (TFA) in dichloromethane (DCM) for 5 × 1 min, followed by washing with DCM and NMP. Compound **7** (5.8 mg, 30 eq), HATU (4.3 mg, 27 eq) were dissolved in 100 µL of NMP and 2 µL of DIPEA and agitated for 1 min. The preactivated mixture was added to the support and shaken for 1 h. The support was filtered and washed with NMP and DCM. The PNA conjugate was cleaved from support by TFA/TIS/water (95/2.5/2.5) (200 µL) for 2 h and the carbohydrate protecting groups were removed using ammonia solution for 5 h at 50 °C. The mixture was then lyophilized and purified as described for **PNA 2** (Retention time **PNA 7**: 15.8 min). Mass calculated for **PNA 7**: C138H183N71O42, 3507; found, [M+] 3508.

### 3.4. Introduction of a New Amino Acid Building Block in the PNA Sequence

*Synthesis of* **PNA-GlyNeo-PNA**. N^β^-methyltrityl protection was cleaved off by subjecting the solid supported PNA-GlyDapa-PNA to 1% trifluoroacetic acid in DCM for 5 × 1 min, followed by washing with DCM and NMP. Then 5-phenoxycarbonylamino-2,9-dimethyl-1,10- phenanthroline (3.1 mg, 9 µmol), NMM (5 µL, 50 µmol) in NMP (75 µL) was added to the support. The reaction was left overnight. After washing with NMP and DCM, the PNA conjugate was cleaved from support by TFA/TIS/water (95/2.5/2.5) (200 µL) for 2 h, freeze dried and purified (Retention time ***PNA-GlyNeo-PNA***: 22.1 min). Mass calculated for ***PNA-GlyNeo-PNA***: C147H183N73O39, 3595; found, [M+] 3597.

*Synthesis of* (**8**). 2-(2-(2-methoxyethoxy)ethoxy]acetic acid (109 µL, 0.7 mmol, 1.2 eq) and HATU (1.1 g, 1.9 eq) were dissolved in 2 mL of DMF and stirred for 2 min in presence of 0.5 eq of NMM and then added to Fmoc-L-Dab-OH (200 mg, 0.58 mmol) previously suspended in 7 mL of DMF. The reaction was run at room temperature for 20 min. The solution was dried and purified using column chromatography on silica eluted with a gradient system running from pure dichloromethane with 1% acetic acid to dichloromethane 90% methanol 15% with 0.1% acetic acid. This gave the product as 181 mg of a yellow oil. Mass calculated for compound 8 [M+], 500.54; found, [M+] 501.6; ^1^H-NMR (CD_3_OD): δ 7.82 (d, 2H, *J* = 7.5); δ 7.71 (t, 2H, *J* = 6.6); δ 7.41 (t, 2H, *J* = 7.4); δ 7.34 (t, 2H, *J* = 7.4); δ 4.40 (d, 2H, *J* = 6.3); δ 4.30–4.19 (m, 2H); δ 4.00 (s, 2H); δ 3.69-3.53 (m, 8H); δ 3.39 (s, 1H); δ 3.35 (s, 3H); δ 3.33 (s, 1H) δ 2.17–2.04 (m, 1H); δ 1.92–1.79 (m, 1H); 13C-NMR (CD_3_OD): δ 173.43; δ 159.10; δ 145.77; δ 129.20; δ 128.60; δ 126.70; δ 121.33; δ 73.26; δ 72.42; δ 71.70; δ 71.58; δ 69.81; δ 68.22; δ 59.46; δ 53.92; δ 48.77; δ 37.16; δ 32.73.

*Synthesis of* **PNA 9**. The terminal Fmoc was cleaved off by subjecting PNA 8 (5 mg, 1.04 µmol) to 20% piperidine in NMP for 25 min, followed by washing several times with DCM and NMP and the terminal amino group was acetylated using a solution of acetic anhydride: lutidine: NMP, 5: 6: 89 (2 × 5 min). The support was filtered and washed with NMP and DCM. N^β^-methyltrityl protection was cleaved off by subjecting the solid support to 1% trifluoroacetic acid in DCM for 5 × 1 min, followed by washing with DCM and NMP then 5-phenoxycarbonylamino-2,9-dimethyl-1,10- phenanthroline (3.1 mg, 9 µmol), NMM (5 µL, 50 µmol) and NMP (75 µL) were added to the support. The reaction was left overnight. The PNA conjugate was cleaved from support by TFA/TIS/water (95/2.5/2.5) (200 µL) for 2 h, freeze dried and purified (Retention time **PNA 9**: 25.5 min). Mass calculated for **PNA 9**: C156H200N74O43, 3798; found, [M+] 3800.

*Synthesis of* **PNA 10**. The terminal Fmoc was cleaved off by subjecting **PNA 8** (5 mg, 1.04 µmol) to 20% piperidine in NMP for 25 min, followed by washing several times with DCM and NMP and then coupled with **PEG 9** (8 mg, 5.8 µmol) in NMP (75 µL) with NMM (50 µmol, 5 µL) was added to the support. The reaction was left overnight. The support was then filtered and washed with NMP and DCM. N^β^-methyltrityl protection was cleaved off by subjecting the solid support to 1% trifluoroacetic acid in DCM for 5 × 1 min, followed by washing with DCM and NMP then 5-phenoxycarbonylamino-2,9-dimethyl-1,10- phenanthroline (3.1 mg, 9 µmol), NMM (50 µmol, 5 µL) and NMP (75 µL) were added to the support. The reaction was left overnight.

The PNA conjugate was cleaved from support by TFA/TIS/water (95/2.5/2.5) (200 µL) for 2 h, freeze dried and purified with a Ascentis Express Supelco Peptide ES-C18 (2, 7 µm 150 × 4.6mm) column at 60 °C using a flow rate of 1 mL/min and a linear gradient of 20%–100% B for 38 min. (A) 0.1% TFA–aq., (B) 0.1% TFA–aq., 50% MeCN (Retention time **PNA 10**: 27.9 min). Mass calculated for **PNA 10**: C210H301N81O66, 5013; found, [M+] 5014.

## 4. Conclusions

We have in this study demonstrated synthesis and approaches to PNA conjugated to different oligoethers, an aminosugar and attachment of one or two different oligoether entities together with a neocuproine derivative. The thermal melting is somewhat affected upon central positioning of the oligoethers but this seems to be at an acceptable level for use of the conjugates. It is less clear how the polarity of the conjugates is affected by addition of oligoethers (as judged from RP-HPLC retention) and it is likely that how and where these are attached as well as the exact nature of the conjugated entities play a role. Conjugation of oligoethers to PNA is in its infancy and is likely to influence solubility and other properties of the PNA, perhaps also catalytic activity of PNAzymes and further studies will reveal such influences. 

## Supplementary Materials

Supplementary materials can be accessed at: http://www.mdpi.com/1420-3049/19/3/3135/s1.
