# Supplementary material for: Synthesis of PNA Oligoether Conjugates
Source: Molecules. 2014 Mar 13;19(3):3135–48. doi: 10.3390/molecules19033135 (PMC6270860; doi:10.3390/molecules19033135)

Supplementary File

Figure S1. ESI-MS (-) and ESI-MS (+) spectra, as well as ^{1}H and ^{13}C-NMR spectra of compound **4**.

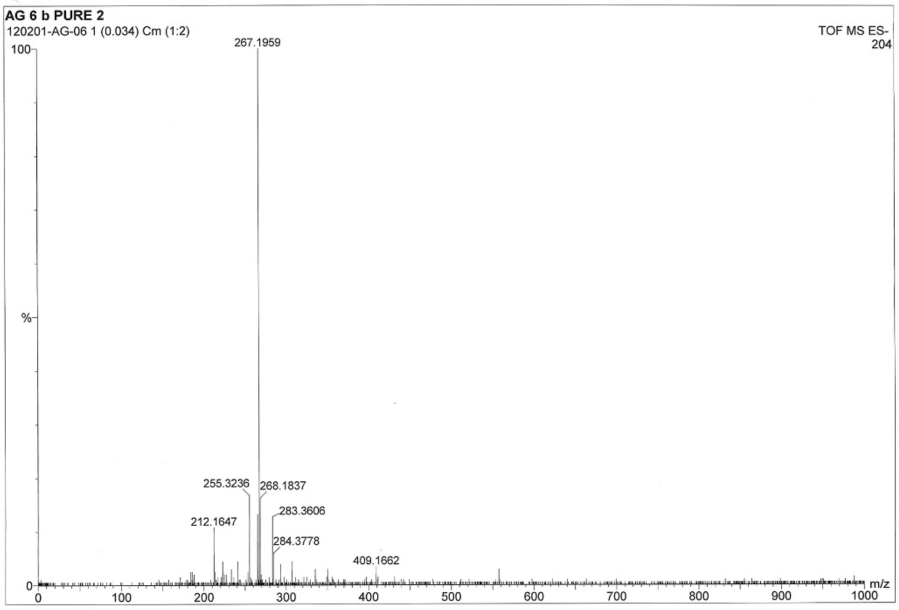

Electrospray time of flight mass spectrum of compound **4** (negative mode)

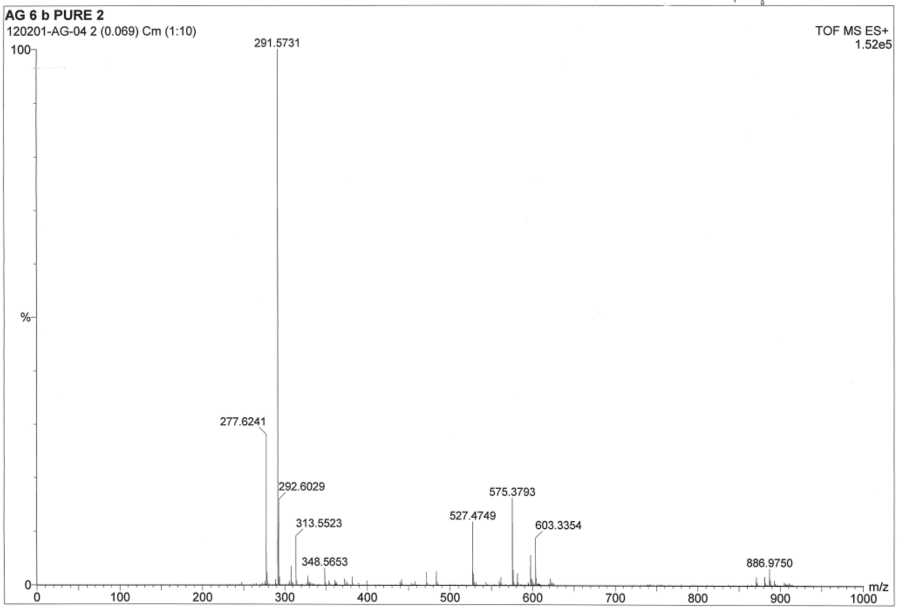

Electrospray time of flight mass spectrum of compound **4** (positive mode)

Figure S1. *Cont.*

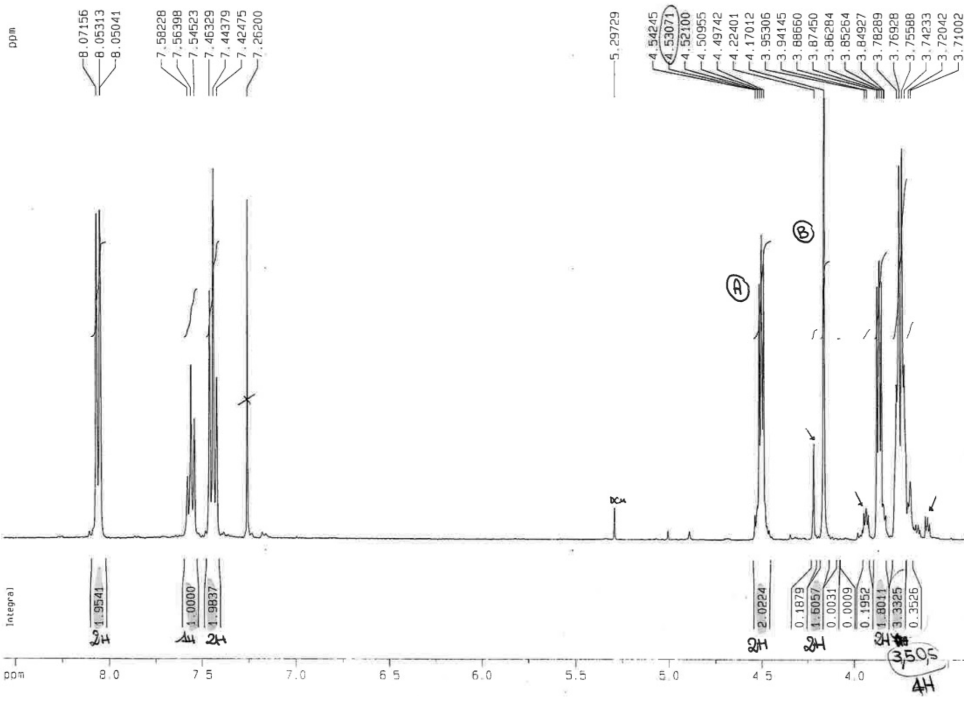

^{1}H-NMR spectrum of compound **4**

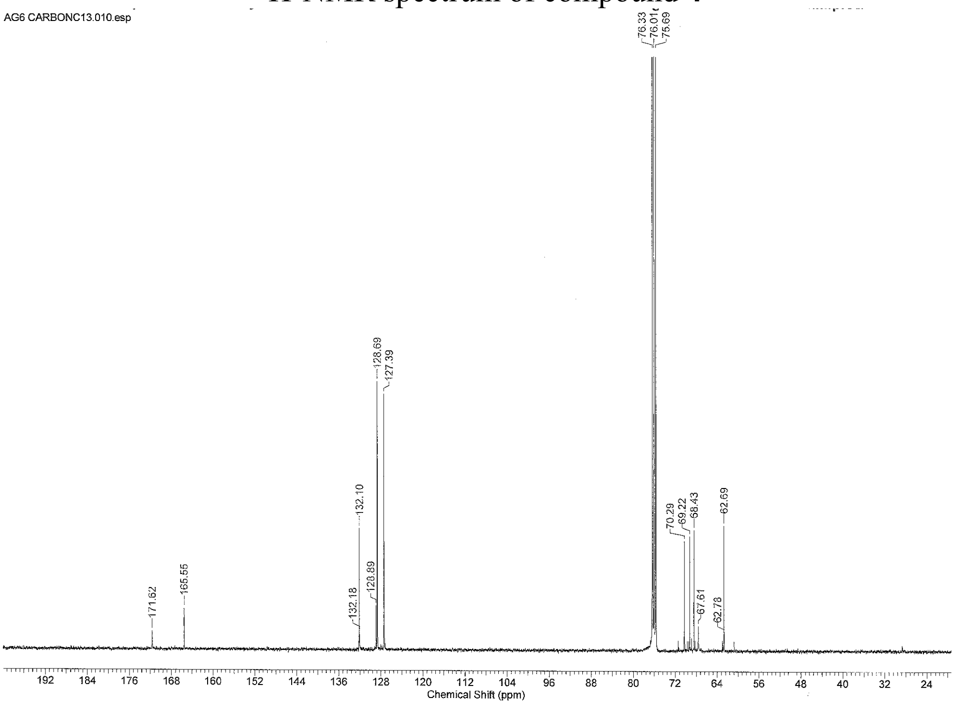

^{13}C-NMR spectrum of compound **4**

Figure S2. ESI-MS, ^{1}H-NMR and ^{13}C-NMR of compound **7**.

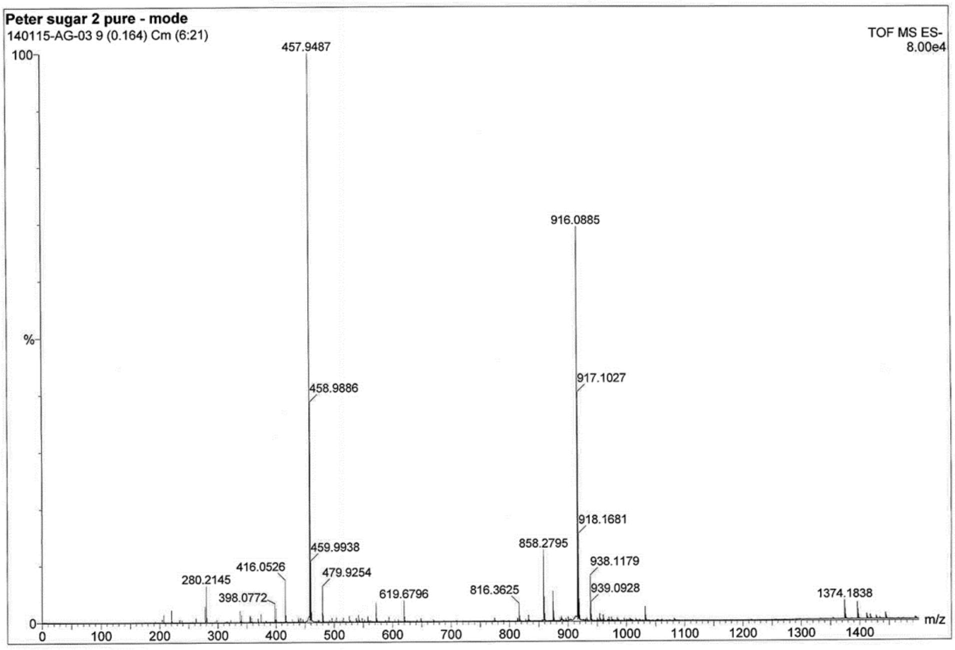

Electrospray time of flight mass spectrum of compound **7** (negative mode)

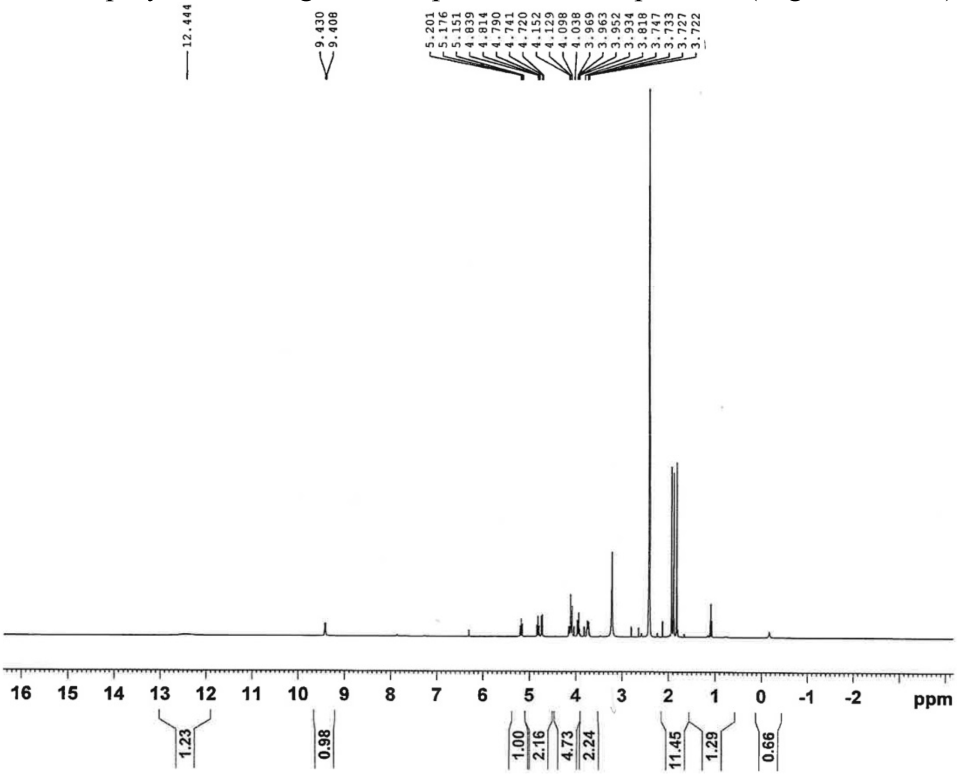

^{1}H-NMR spectrum of compound **7**

Figure S2. *Cont.*

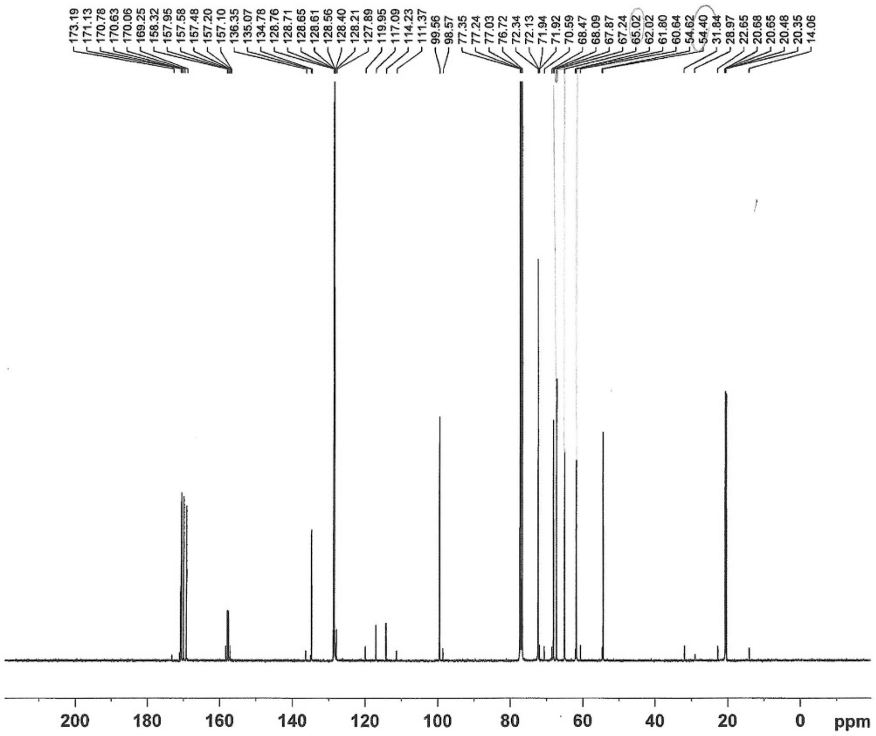

^{13}C-NMR spectrum of compound **7**

Figure S3. ESI-MS, ^{1}H-NMR and ^{13}C-NMR of compound **8**.

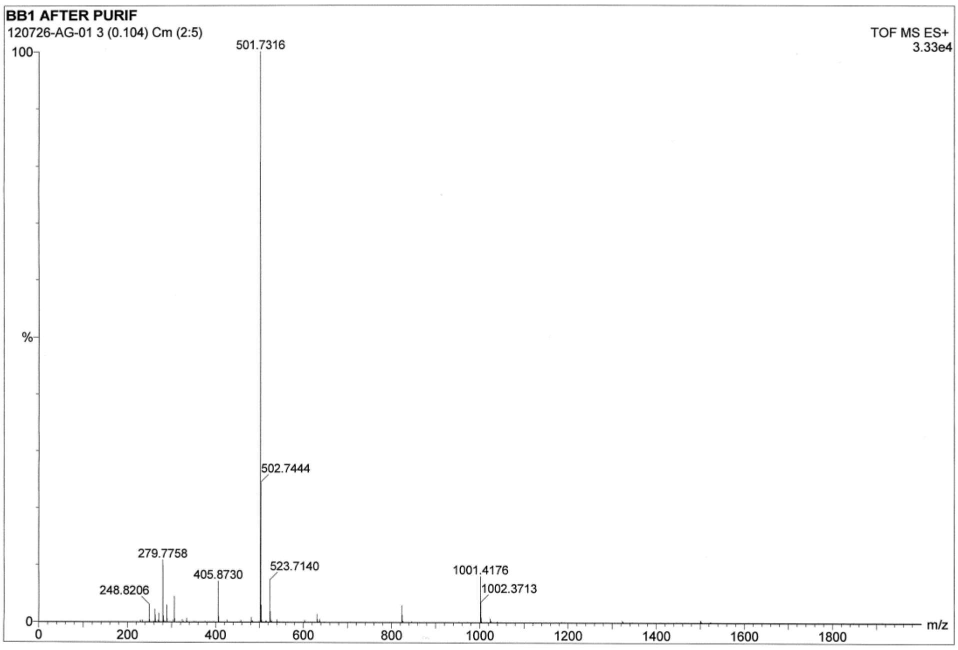

Electrospray time of flight mass spectrum of compound **8** (positive mode)

Figure S3. *Cont.*

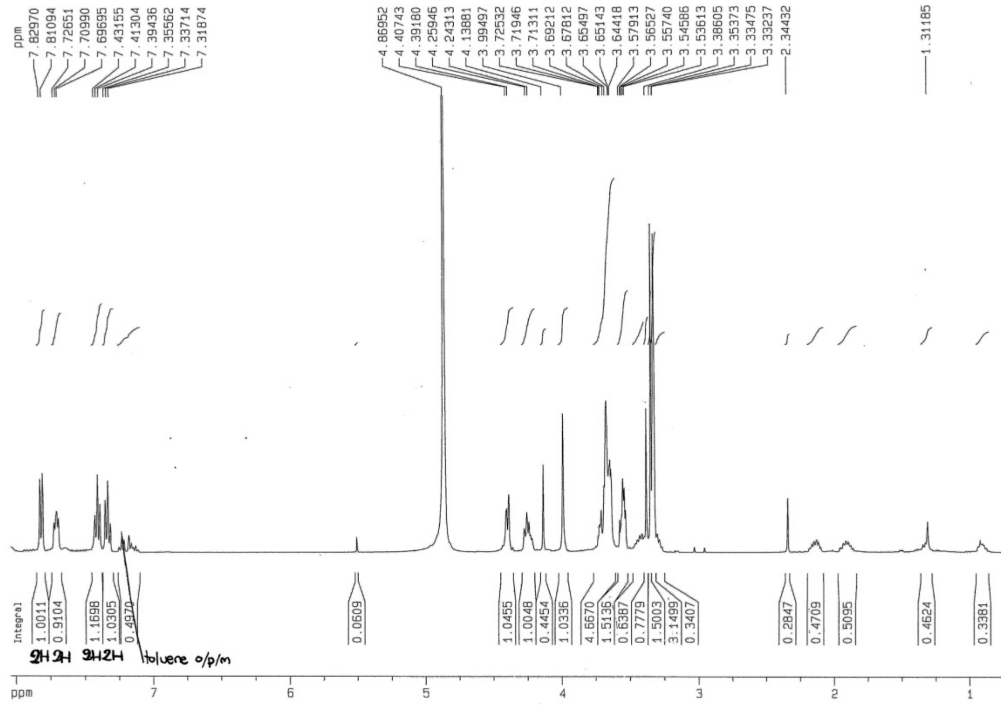

^{1}H-NMR spectrum of compound **8**

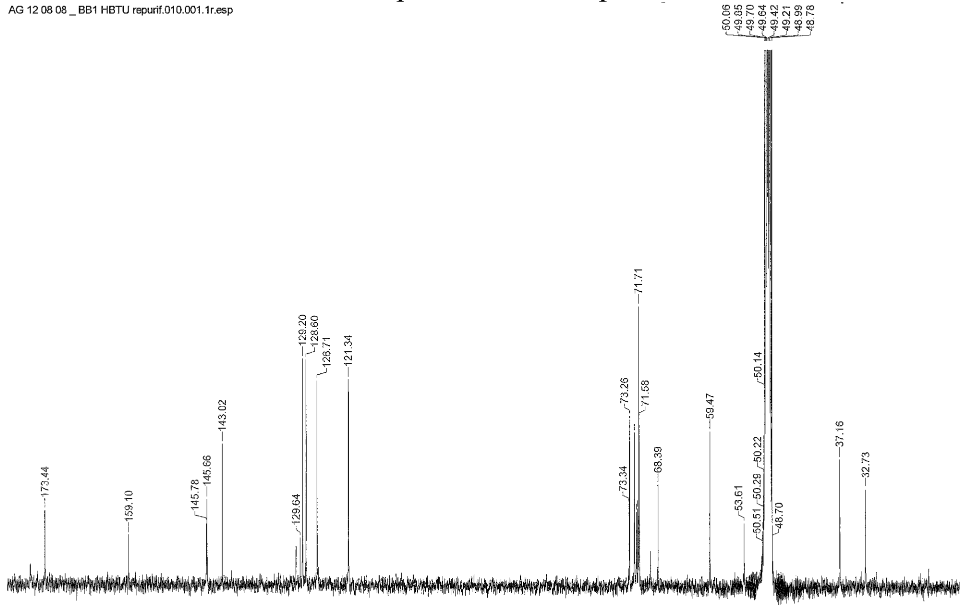

^{13}C-NMR spectrum of compound **8**

Figure S4. Chromatograms of crude (left panels) and purified PNAs (right panels) and ESI-MS of purified PNA-conjugates (lower panels).

PNA 2

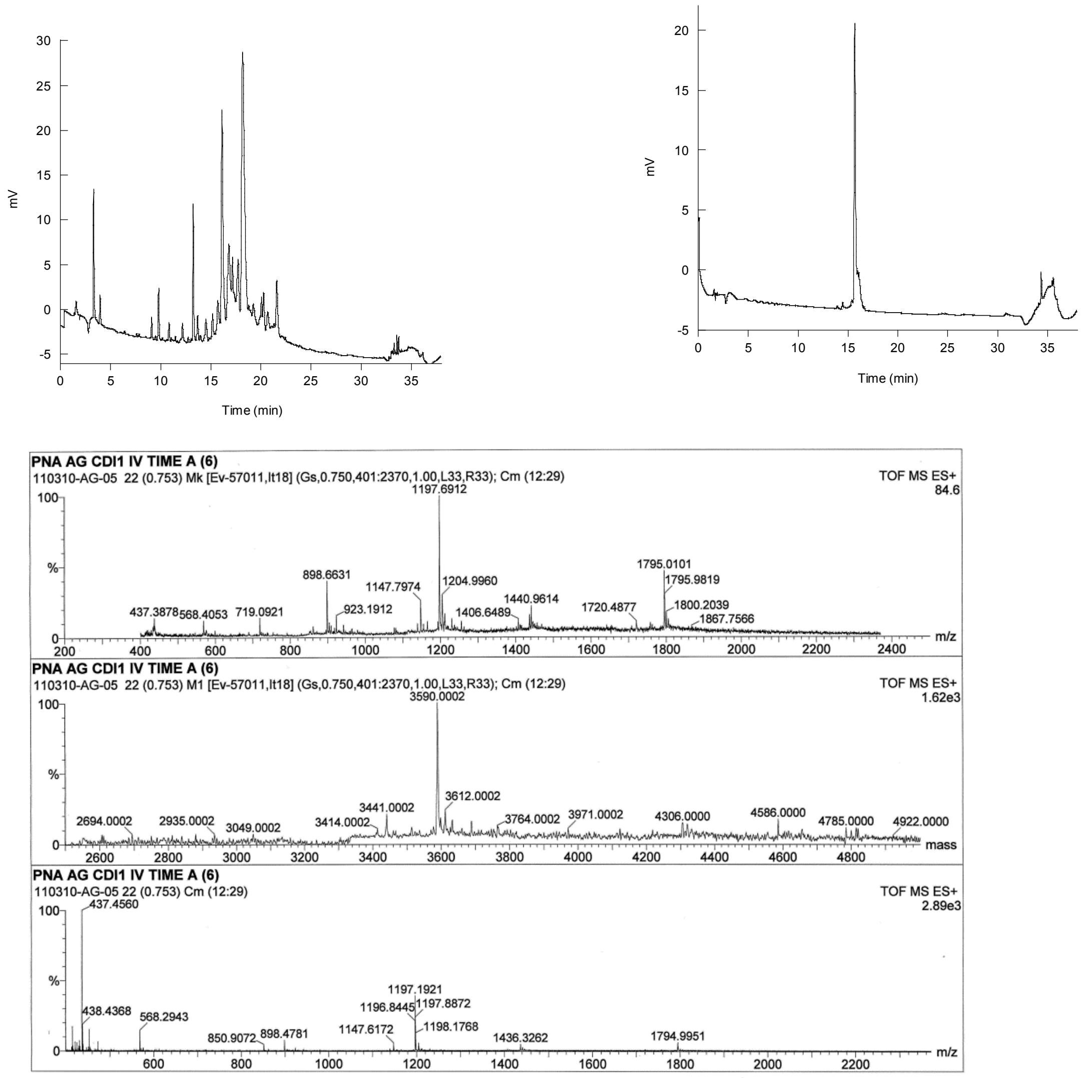

PNA 3

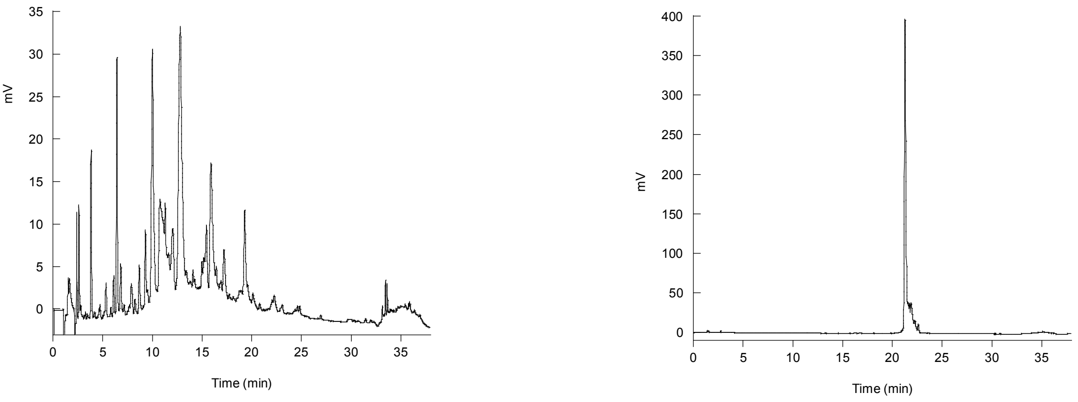

Figure S4. *Cont.*

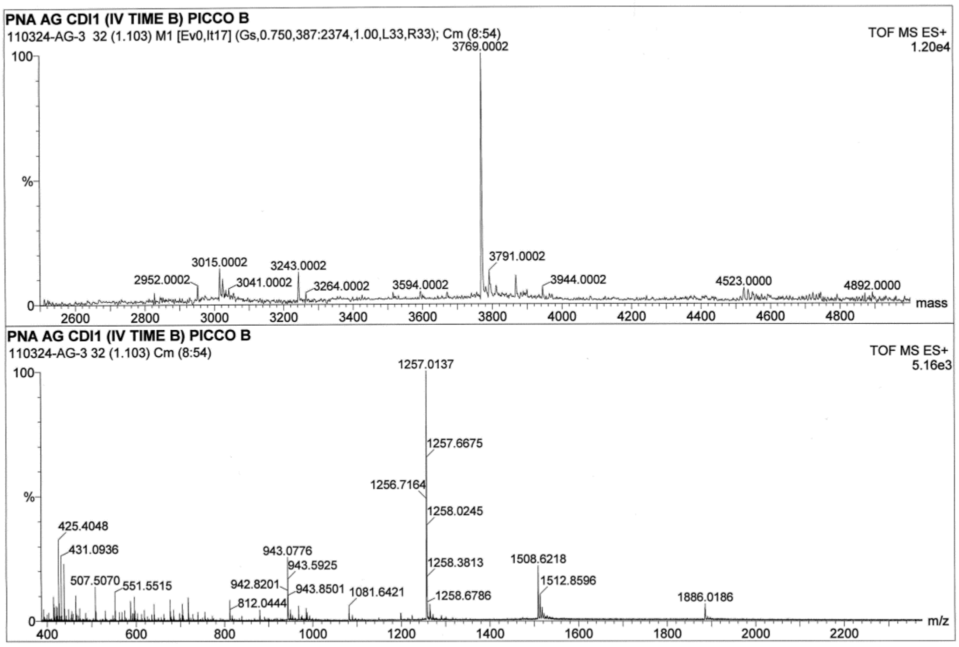

PNA 5

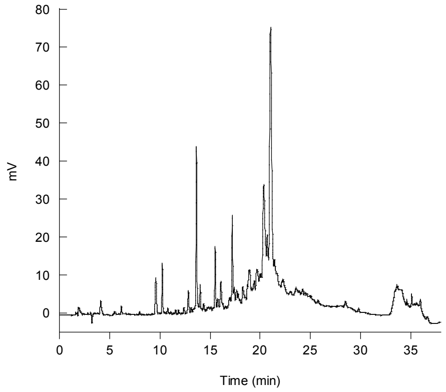

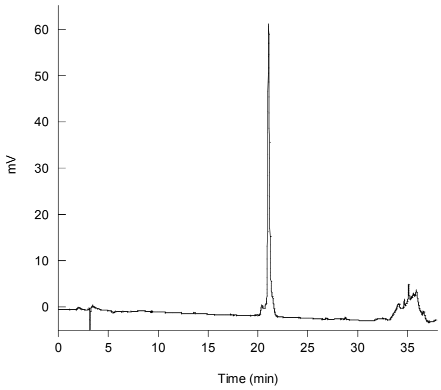

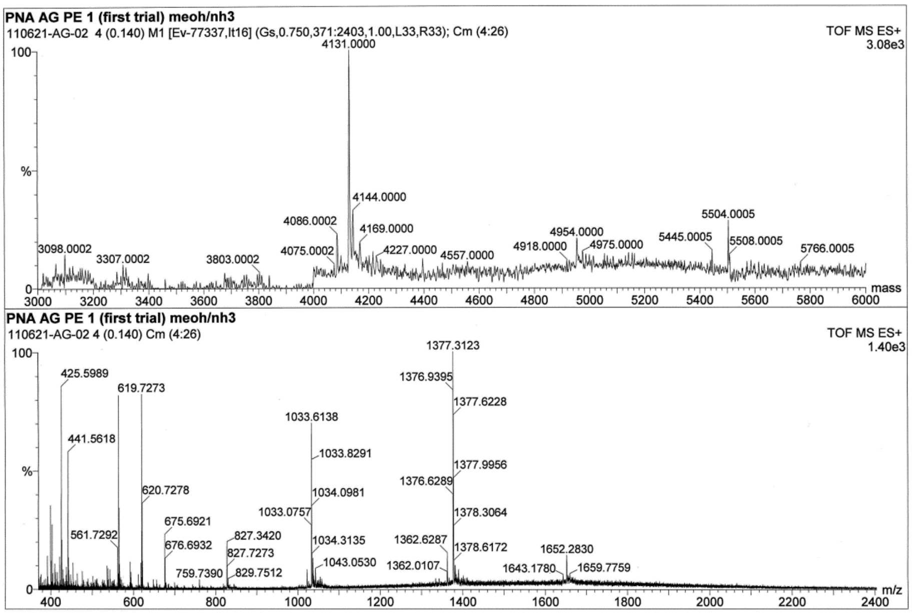

Figure S4. *Cont.*

PNA 7

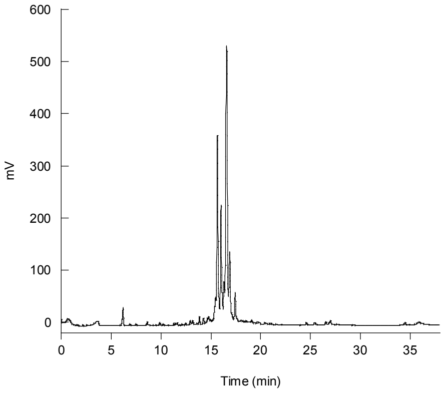

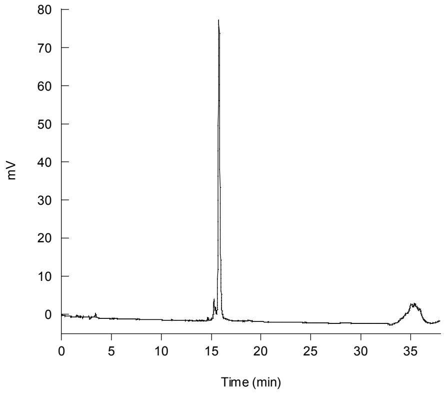

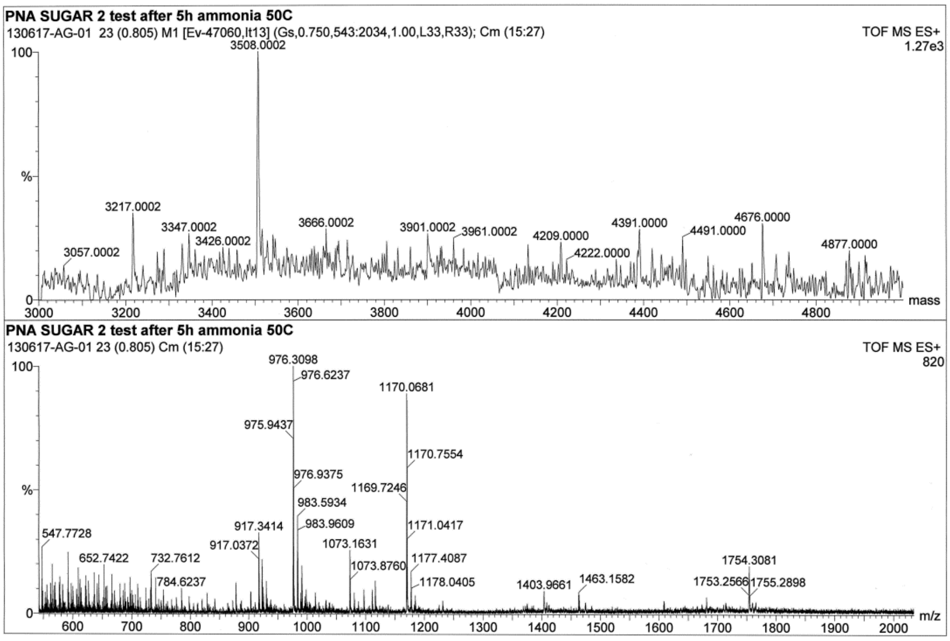

PNA 9

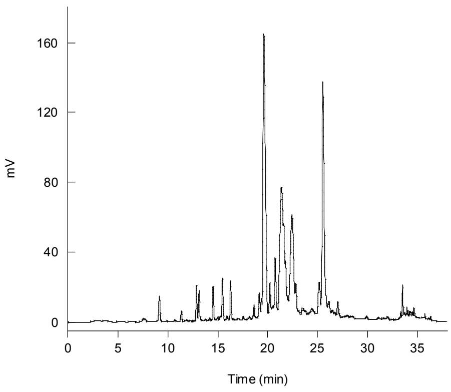

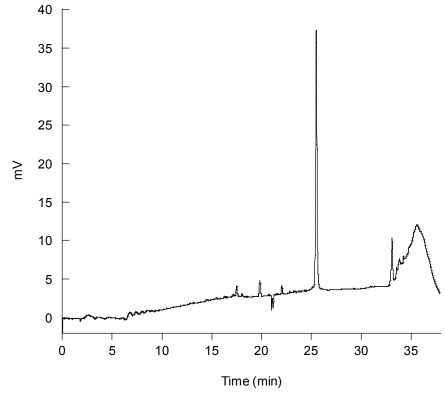

Figure S4. *Cont.*

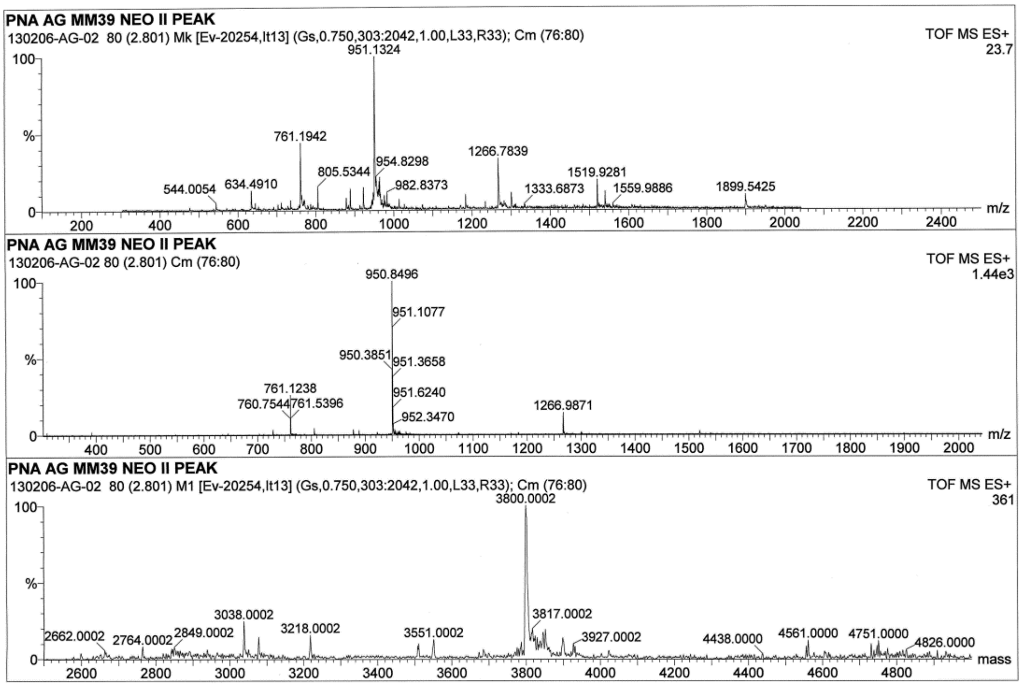

PNA 10

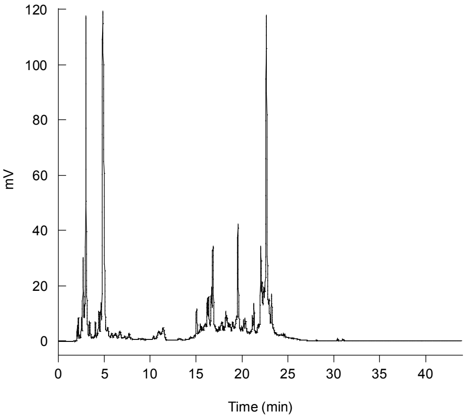

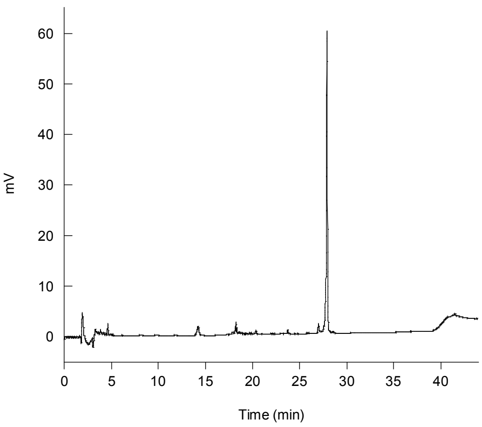

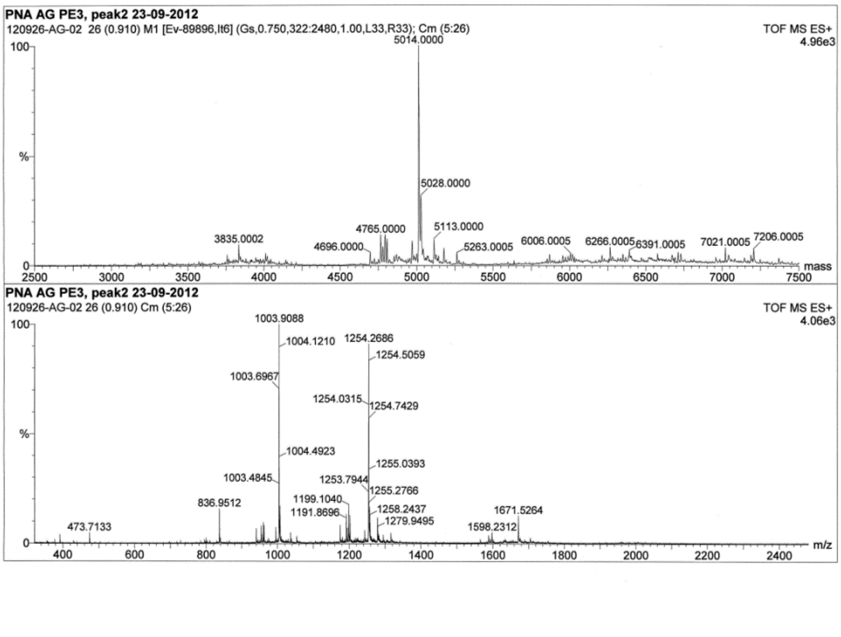

Supplement: Supplementary file 1 [file molecules-19-03135-s001.pdf]
